# Prognostic Value of Inflammatory Markers in Septic Critically Ill Patients with Chronic Liver Disease: A Retrospective Analysis*

**DOI:** 10.5152/tjg.2025.24794

**Published:** 2025-06-16

**Authors:** Nazlıhan Boyacı Dündar, Kamil İnci, Gülbin Aygencel, Melda Türkoğlu, Onur Gökçe, Mehmet Cindoruk

**Affiliations:** 1 Department of Internal Medicine, Division of Intensive Care Medicine, Gazi University School of Medicine, Ankara, Türkiye; 2 Yunus Emre State Hospital, Eskişehir, Türkiye; 3 Department of Gastroenterology and Hepatology, Gazi University School of Medicine, Ankara, Türkiye

**Keywords:** Chronic liver disease, CLIF-C ACLF score, inflammatory markers, lactate-to-albumin ratio, mortality, neutrophil-to-lymphocyte ratio, sepsis

## Abstract

**Background/Aims:** Septic patients with chronic liver disease (CLD) experience high morbidity and mortality rates, particularly in the intensive care unit (ICU) setting, due to immune dysfunction. Despite their vulnerability, data on prognostic markers remain scarce, particularly when assessed in conjunction with disease severity scores. This study aimed to evaluate the prognostic value of various inflammatory markers, including white blood cell count (WBC), neutrophil-to-lymphocyte ratio (NLR), lactate, and lactate-to-albumin ratio (LAR), in septic critically ill CLD patients.**Materials and Methods:** A retrospective cohort study was conducted on 126 septic CLD patients admitted to ICU. Data on demographics, clinical scores, inflammatory markers, and clinical outcomes were collected. Logistic regression and ROC analyses were used to identify independent predictors of ICU mortality.**Results:** Intensive care unit mortality was 66%. In addition to higher Acute Physiology and Chronic Health Evaluation II (APACHE II) (39.3 ± 7.2 vs. 21 ± 5.1, *P* < .001), Sequential Organ Failure Assessment (12.4 ± 3.5 vs. 8.5 ± 3.1,* P* < .001), CLIF-C ACLF [63 (54-69) vs. 50 (41-53)] scores, ICU non-survivors had higher WBC (median: 14 400/µL vs. 7300/µL,* P* < .001), lactate (median: 4.6mmol/L vs. 2.4mmol/L, *P* < .001), NLR (median: 12.5 vs. 9, *P* = .015), and LAR (median: 2.15 vs. 0.93, *P* < .001) compared to survivors. Multivariate analysis identified APACHE II (OR 1.183, 95% CI: 1.003-1.396, *P* = .046), CLIF-C ACLF (OR 1.104, 95% CI: 1.002-1.216, *P* = .046), and LAR (OR 2.992, 95% CI: 1.277-7.009, *P* = .012) as independent predictors of ICU mortality. The LAR was the most significant inflammatory marker (area under the curve: 0.783, cut-off: 1.17), even in the subgroup of patients with low acute decompensation scores based on the CLIF-C ACLF score.**Conclusion:** The LAR was a valuable prognostic marker for ICU mortality in septic CLD patients, even in the absence of advanced organ failure. This marker potentially outperforms other traditional inflammatory markers and could aid in early risk stratification for critically ill septic CLD patients.

**Main Points**

- The prognostic utility of inflammatory markers, including the white blood cell count, neutrophil-to-lymphocyte ratio (NLR), derived NLR, platelet-to-lymphocyte ratio, platelet-to-creatinine ratio, lactate, lactate-to-albumin ratio (LAR), C-reactive protein, and procalcitonin, in predicting outcomes among critically ill septic patients with chronic liver disease (CLD) was assessed.
- The Acute Physiology and Chronic Health Evaluation II score, Chronic Liver Failure-Consortium Acute-on-Chronic Liver Failure score, and LAR were independent predictors of intensive care unit (ICU) mortality in critically ill septic patients with CLD.
- Baseline LAR value was independently associated with increased ICU mortality, even in the absence of advanced organ failure, providing valuable prognostic insights into the clinical course of sepsis in this vulnerable population.

## Introduction

Sepsis, a life-threatening condition driven by an uncontrolled immune response to infection, leads to particularly poor outcomes in patients with chronic liver disease (CLD).^1^ Individuals with CLD face an increased risk of infection due to immune dysfunction, bacterial translocation, and impaired hepatic clearance of endotoxins.^2^ Due to these factors, this patient group is at high risk of sepsis, which results in significant morbidity and mortality, particularly in cases requiring intensive care unit (ICU) admission.^3^^,^^4^ Despite modern critical care interventions, the mortality rate for septic patients with CLD remains alarmingly high, with ICU mortality reaching up to 67%.^4^^,^^5^

Recently, there has been a growing focus on using inflammatory markers as predictive tools for outcomes in patients with sepsis. In addition to traditional inflammatory markers such as white blood cell count (WBC), polymorphonuclear leukocytes (PMNL), C-reactive protein (CRP), and procalcitonin (PCT), numerous novel biomarkers and derived calculations are being developed to assess their potential in predicting prognosis more simply and effectively in this patient population.^6^^,^^7^^,^^8^ While commonly studied markers like the CRP, PCT, neutrophil-to-lymphocyte ratio (NLR), and lactate-to-albumin ratio (LAR) have demonstrated potential in reflecting sepsis severity and predicting outcomes, research has predominantly focused on the general septic population.^6^^,^^7^^,^^8^ Despite the fact that patients with CLD exhibit a persistent inflammatory state and altered immune responses, studies specifically investigating inflammatory markers in this subgroup remain scarce, highlighting a gap in the literature.^2^^,^^9^ Recently, the association between LAR and both short- and long-term adverse outcomes has been demonstrated in a large cohort of septic cirrhotic patients; however, its relationship with routinely used inflammatory markers in this population remains unclear.^10^^,^^11^

This study seeks to fill this gap by assessing the prognostic value of various inflammatory markers and their relationship with clinical outcomes, such as ICU mortality rates, duration of ICU stay, and disease severity in this vulnerable patient population. Moreover, this study also aims to compare the prognostic value of these markers with established disease severity scores such as the Acute Physiology and Chronic Health Evaluation II (APACHE II), Sequential Organ Failure Assessment (SOFA), Model for End-Stage Liver Disease (MELD), Child-Pugh and Chronic Liver Failure-Consortium Acute-on-Chronic Liver Failure (CLIF-C ACLF) scores, which may guide novel research regarding risk stratification in septic critically ill patients with CLD.

## Materials and Methods

### Study Design and Setting

This retrospective cohort study was conducted in the tertiary, 9-bed medical ICU of Gazi University University Hospital, covering the period from January 2012 to January 2024. The research protocol complied with the Declaration of Helsinki and was approved by the Local Ethics Committee of Gazi University University (Approval Number: 2024-982 and Date: June 11, 2024). Since the data were obtained retrospectively from existing records, informed consent was not required.

### Participants

The study included adult patients aged 18 and above with a confirmed diagnosis of CLD who were admitted to the ICU due to sepsis, as defined by the Sepsis-3 criteria.^12^Exclusion criteria were: ICU stays shorter than 24 hours, sepsis diagnosed more than 24 hours prior to admission for patients transferred from other hospitals, liver failure due to neoplastic metastasis, terminally ill patients, and ICU admissions solely for post-transplantation follow-up. Only the first septic hospitalization period was analyzed for patients with multiple ICU admissions.

### Data Collection

Data were extracted from electronic medical records and hospital archives, including demographics, prior hospitalizations, Glasgow Coma Scale score, and clinical scores: APACHE II, SOFA, MELD, Child-Pugh, and CLIF-C ACLF. Information on comorbidities, the requirements for mechanical ventilation, vasopressors, renal replacement therapy, blood transfusion, and albumin replacement as well as length of ICU stay and ICU mortality, were also recorded. Laboratory data, including inflammatory markers CRP, PCT, WBC, PMNL, NLR (measured by dividing the number of neutrophils by the number of lymphocytes), derived NLR (dNLR, the ratio of neutrophils to the difference between total leukocytes and neutrophils in peripheral blood), platelet-to-lymphocyte ratio (PLR, the ratio of platelet to the number of lymphocytes), LAR [measured by dividing the lactate (mmol/L) by the concentration of serum albumin (g/dL)] and platelet-to-creatinine ratio [PCR; the ratio of platelet to the concentration of serum creatinine (mg/dL)] were collected at ICU admission. The primary outcome of the study was ICU mortality.

### Statistical Analysis

Continuous variables are presented as means ± SD or medians with interquartile ranges, depending on the data distribution. Categorical variables are expressed as frequencies and percentages. Patients were divided into 2 groups, survivors and non-survivors, based on ICU mortality, and comparisons were made using the Mann-Whitney *U* test or Independent- samples T-test for continuous variables and the chi-squared test for categorical variables. Logistic regression analysis was performed to identify independent predictors of ICU mortality. The AUCs were calculated by plotting ROC curves to determine the relationship between independent prognostic factors and ICU mortality. A *P*-value <.05 was considered statistically significant. All statistical analyses were conducted using the SPSS software program version 22.0 (IBM SPSS Corp.; Armonk, NY, USA).

## Results

Baseline characteristics and complications related to liver disease on ICU admission of 126 septic ICU patients with CLD are given in Table 1. Among these patients, overall ICU mortality was 66% (83 patients). The APACHE II, SOFA, and CLIF-C ACLF scores were significantly higher among ICU non-survivors (APACHE II: 39.3 ± 7.2 vs. 21±5.1, SOFA: 12.4 ± 3.5 vs. 8.5 ± 3.1, CLIF-C ACLF [63 (54-69) vs. 50 (41-53)] scores *P* < .001 for both), indicating greater severity of illness (Table 1). Moreover, the MELD score was significantly higher in non-survivors compared to survivors [28 (23-34) vs. 23 (19-29), *P* = .002] (Table 1). Hepatic encephalopathy (HES) was the most common complication related to liver disease on ICU admission (42.8%), and respiratory infections were the most common source of sepsis, particularly prevalent among non-survivors (44.5% vs. 23.2%, *P* = .02) (Table 1).

Baseline laboratory findings according to ICU mortality are given in Table 2. Non-survivors had higher WBC (median: 14  400/µL vs. 7300/µL, *P* < .001), higher PMNL (median: 11  200/µL vs. 6100/µL, *P* < .001), higher lactate levels (median: 4.6 mmol/L vs. 2.4 mmol/L, *P* < .001), higher NLR (median: 12.5 vs. 9, *P* = .015), higher CRP (median: 89 mg/L vs 54 mg/L, *P* = .03), and higher LAR (median: 2.15 vs. 0.93, *P* < .001).

**Figure 1.**
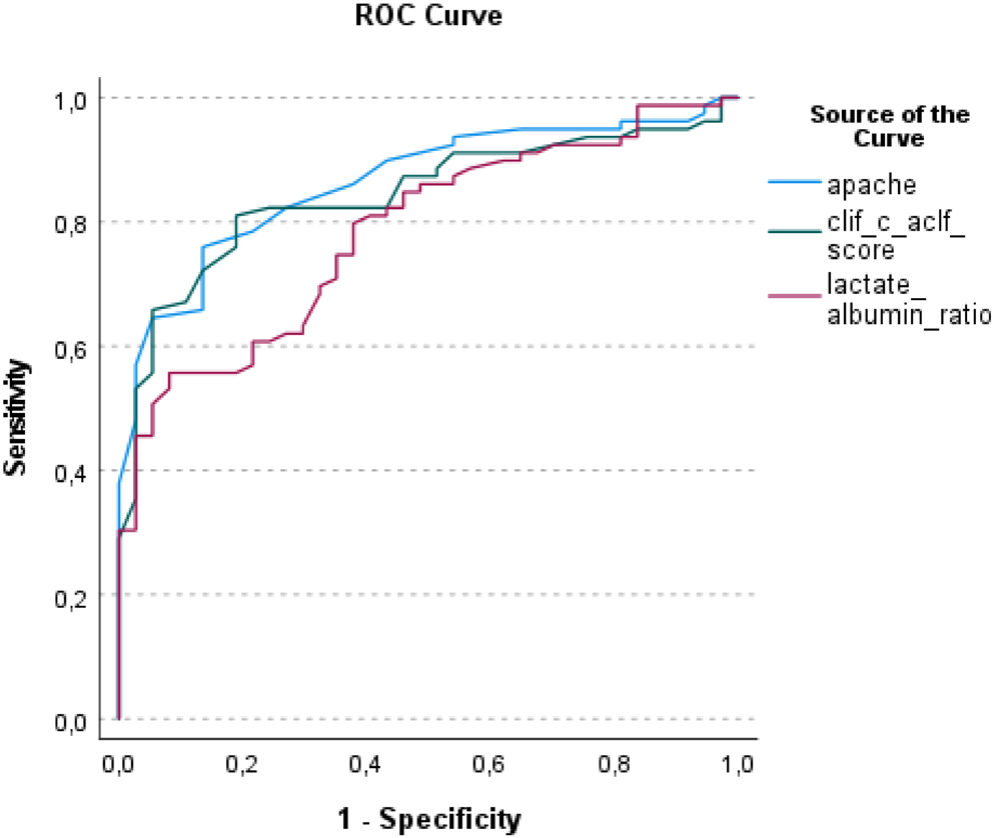
ROC curve of APACHE II, CLIF-C ACLF Score and LAR (CLIF-C ACLF, Chronic Liver Failure-Consortium Acute-on-Chronic Liver Failure; APACHE, Acute Physiology and Chronic Health Evaluation; AUC, area under the curve, LAR, lactate albumin ratio).

Multivariate analysis identified several independent predictors of ICU mortality, including a higher APACHE II score (OR 95%CI: 1.183 [1.003-1.396], *P* = .046), a higher CLIF-C ACLF score (OR 95%CI: 1.104 [1.002-1.216], *P* = .046), and elevated LAR (OR 95%CI: 2.992 [1.277-7.009], *P* = .012) (Table 3). The APACHE II and CLIF-C-ACLF score showed similar significance (cut-off value: 24.5, sensitivity: 82%, specificity: 73%, AUC: 0.864, cut-off value: 53.5, sensitivity: 82%, specificity: 81%, AUC: 0.843, respectively), while LAR was the most significant biomarker in predicting ICU mortality (cut-off value: 1.17, sensitivity: 81%, specificity: 61%, AUC: 0.783) (Figure 1).

Subgroup analysis was performed to evaluate whether LAR and other inflammatory markers could be useful in identifying high-risk septic CLD patients without advanced organ failure. Only patients with low acute decompensation scores based on the CLIF-C ACLF score were included in the analysis. Patients with a CLIF-C ACLF score greater than 53, derived from the significant mortality threshold based on the ROC curve in the main cohort, were excluded. In the subgroup analysis of 55 patients, the mortality rate was 38.2%. Univariate analysis revealed that, except for LAR, other inflammatory markers were not significantly associated with mortality (Supplementary Tables 1 and 2 in the supplemental file). Multivariate analysis identified respiratory infection as the source of sepsis (OR 95%CI: 8.689 (1.036-72.839), *P* = .032) and elevated LAR (OR 95%CI: 3.768 (2.120-12.682), *P* = .032) as independent predictors of ICU mortality (Supplementary Table 3 in the supplemental file). Even in this subgroup, LAR demonstrated similar sensitivity and specificity at the same cut-off value cut-off value: 1.17, Sensitivity: 79% Specificity: 63%, AUC: 0.777, *P* = .001) (Supplementary Figure 1 in the supplemental file).

## Discussion

In this study, the prognostic utility of inflammatory markers was investigated, including the WBC, PMNL, NLR, dNLR, PLR, PCR, lactate, LAR, CRP, and PCT in predicting outcomes among septic patients with CLD admitted to a medical ICU in a university hospital. These findings suggest that in addition to APACHE II, SOFA, and CLIF-C-ACLF scores, WBC, PMNL, CRP, lactate, NLR, and LAR were higher in ICU non-survivors than survivors. The incorporation of the CLIF-C ACLF score provided additional insights into the prognosis of septic patients with CLD, underlining its value in assessing organ dysfunction severity specific to this population. Furthermore, the baseline LAR value was independently associated with increased ICU mortality, even in the subgroup of patients with acute decompensation without advanced organ failure, highlighting its critical role in identifying high-risk patients and guiding clinical decision-making in this vulnerable group.

The unique immunopathology of CLD, characterized by immune dysfunction, bacterial translocation, and impaired endotoxin clearance, increases the susceptibility of these patients to severe sepsis and multi-organ failure.^2^^,^^13^^,^^14^ It is well defined that inflammatory response is a significant factor in the mechanisms related to the progression, decompensation, and complications of CLD.^3^^,15^ This altered immune landscape is reflected in elevated NLR levels, a marker of systemic inflammation, which represents the balance between neutrophil-mediated innate immunity and lymphocyte-driven adaptive regulation.^16^ The ratio has shown prognostic value due to its ease of calculation from routine blood tests and because it reflects the balance between neutrophilic immune response and lymphocyte-mediated regulation even in cirrhotic patients.^17^ Magalhaes et al^18^ have recently demonstrated that higher NLR values at admission were associated with infection risk in cirrhotic patients. Although NLR was not independently associated with ICU mortality in patients with CLD in this study, it was significantly higher in ICU non-survivors compared to survivors. When considered alongside the current literature, these findings suggest that in septic CLD patients, NLR is more closely related to the presence of infection rather than the severity of the disease.^17^^,^^18^

CRP and PCT are known to be traditional inflammatory markers commonly used in clinical practice to assess infections and predict prognosis.^19^ However, in advanced liver disease, the clinical utility of these 2 markers is associated with contradictory results in the existing literature.^19^^-^^22^The baseline CRP and PCT levels can be elevated even in the absence of infection.^20^^,^^22^^-^^25^Recently, in a study conducted by Liu et al^19^ aiming to establish a scoring model for the early diagnosis of infection, they demonstrated significantly higher levels of PCT, CRP, neutrophil percentage, and lactate in the infection group, which predominantly included patients with acute-on-chronic liver failure. In the multiple regression analysis, PCT, CRP, and lactate retained their significance for the diagnosis of infection.^19^ The difference between the study and the aforementioned one lies in evaluating the relationship between traditional inflammatory markers and clinical outcomes in septic CLD patients. In this patient group, in which CRP and PCT synthesis is expected to be impaired, it was evaluated whether there is a threshold value that could alert clinicians regarding adverse clinical outcomes. In this context, WBC, PMNL, NLR, CRP, and lactate levels were higher in ICU non-survivors than in survivors; however, none of these were found to be independent risk factors for ICU mortality. Furthermore, no significant difference in those parameters was found in the subgroup analysis of patients without advanced organ failure. This suggests that although these inflammatory markers, commonly used to assess impaired immune response in CLD, are frequently utilized in the early detection of adverse prognoses leading to organ failures, they are not clinically significant in identifying poor outcomes in the early phase of sepsis. Given the complexity of previous literature data, the single-center experience should be interpreted with caution and validated in larger populations.

Moreover, the findings underscore the potential of LAR as a novel prognostic marker in septic patients with CLD. In recent years, LAR has gained popularity as a biomarker for predicting prognosis in critically ill patients. It has been shown to be an independent risk factor for mortality both in general ICU populations and in specific disease groups, with a reported cut-off range of 0.71 to 1.15 across different patient cohorts.^8^^,^^26^^-^^29^ This study also found LAR to be an independent risk factor and the most significant marker in predicting ICU mortality, with an AUC of 0.783 and a cut-off value of 1.17. In 2 large retrospective cohort studies published very recently, LAR was identified as an independent risk factor for both short-term and long-term mortality in septic cirrhotic patients.^10^^,^^11^ Notably, both studies were conducted using the same retrospective cohort derived from the MIMIC-IV database, resulting in remarkably similar findings.^10^^,^^11^ Their reported cut-off values (1.0 and 1.05, respectively) are consistent with the findings of this study. In the first study, Wang et al^10^ reported a slightly lower AUC (63.34%) in predicting the risk of 28-day all-cause mortality compared to this study. This discrepancy may be attributed to the significantly higher SOFA scores in the study population, which are associated with a higher ICU mortality rate.In the second study conducted by Ma et al,^11^ similar to the study, both LAR and the SOFA and CLIF-C ACLF scores were assessed together. It was found that LAR is an independent risk factor for adverse clinical outcomes in septic cirrhotic patients, regardless of the presence of acute decompensation. An important aspect of the study is that it demonstrates the applicability of the results of these 2 large-scale studies to septic CLD patients in a different geographical region.^10^^,^^11^

This study also aligns with prior findings that underscore the prognostic value of scoring systems like APACHE II, SOFA, and CLIF-C ACLF scores in septic patients with CLD.^30^^-34^ The integration of ACLF scores into the analysis added an extra layer of understanding. Higher scores were significantly associated with increased mortality, consistent with organ failures.^30^^-^^32^ The high mortality rates observed in the cohort, despite advances in ICU care, reflect the higher severity of illness in the cohort and persistent challenges in managing septic patients with CLD, highlighting the need for early identification of those at the highest risk. In this study, the subgroup analysis of patients with low CLIF-OF ACLF scores revealed that similar sensitivity and specificity were achieved with the same cut-off value. This finding is particularly meaningful as it highlights the potential of LAR in predicting poor outcomes, even in septic CLD patients before advanced organ failure occurs. This is a valuable example of the clinical translation of the findings from the previous study conducted by Ma et al.^11^ Ma et al^11^ have recently demonstrated that the LAR combined with the CLIF-OF ACLF score (CLIF-OF ACLF_LAR_) in predicting the mortality exhibited marginal increases to the CLIF-OF ACLF score. Compared to the CLIF-OF ACLF_LAR_ score, which involves a logarithmic calculation, recognizing that LAR, as a simple bedside calculation is typically above 1.1, will provide clinicians with significant ease in the early identification of high-risk patients.^10^^,^^11^

Although the primary strength of this study lies in its comprehensive evaluation of the impact of various inflammatory markers and disease severity scores—many of which have been frequently highlighted and emphasized in the current literature—on ICU mortality in septic patients with CLD, it has several limitations, including its retrospective nature and single-center design, which may limit the generalizability of the findings. Additionally, certain confounding variables were not explored, such as blood product and albumin transfusions prior to ICU admission, type and amount of fluid therapy, and fluid balance, which can impact lactate and albumin levels. Due to the retrospective nature of the study, the lack of details regarding whether appropriate antibiotic therapy was initiated in a timely manner, vasopressor titration, and the administration of hydrocortisone, all of which could directly impact prognosis in septic patients, is also among the major limitations of the study.

In conclusion, this study demonstrates that LAR is potentially a valuable prognostic marker for ICU mortality in septic patients with CLD, even in the absence of advanced organ failure. Compared to various complex disease severity scores, recognizing that the LAR value, obtained through a simple bedside ratio calculation, is typically above 1.1, will provide clinicians with significant ease in the early identification of high-risk patients. Further studies integrating these markers with conventional prognostic scores may enhance early risk assessment, offering a more tailored approach to managing this vulnerable population.

## Supplementary Materials

Supplementary Material

## Figures and Tables

**Table 1.** Comparison of Baseline Characteristics According to Intensive Care Unit Outcome in Septic Patients with chronic Liver Disease

	All Patients (n = 126)	Survivors (n = 43)	Non-Survivors (n = 83)	P**
Age (mean ± SD)	61.1 ± 14.7	61.2 ± 13.2	61 ± 15.5	.468
Gender, n (%)				
Female	50 (39.6)	21 (48.8)	29 (34.9)	.09
APACHE II Score (mean ± SD)	27.1 ± 7.9	21 ± 5.1	39.3 ± 7.2	**<.001**
SOFA Score (mean ± SD)	11 ± 3.8	8.5 ± 3.1	12.4 ± 3.5	**<.001**
Glasgow Coma Scale*	12 (8-14)	14 (12-15)	10 (7-13)	**<.001**
Length of ICU stay (day)*	4 (2-12)	4 (3-8)	5 (2-14)	.81
MELD Score*	26 (22-31)	23 (19-29)	28 (23-34)	**.002**
ACLF Grade n (%)				**<.001**
I	39 (31)	25 (58.1)	14 (16.9)	
II	39 (31)	8 (18.6)	31 (37.3)	
II	37 (29.4)	0	37 (44.6)	
CLIF-C ACLF Score*	57 (49-66)	50 (41-53)	63 (54-69)	**<.001**
Child–Pugh Score*	10 (9-13)	10 (8-11)	11 (9-12)	**.003**
Child–Pugh, n (%)				.127
A	3 (2.4)	2 (4.7)	1 (1.2)	
B	39 (31)	17 (39.6)	22 (26.5)	
C	84 (66.7)	24 (55.9)	60 (72.3)	
Shock on ICU admission, n (%)	72 (57.1)	13 (30.2)	59 (70.1)	**<.001**
Respiratory failure on admission, n (%)	31 (24.6)	7 (16.2)	24 (28.9)	.09
**Complication Related to Liver Disease on Admission, n (%)**				
HRS	35 (27.7)	13 (30.2)	22 (26.5)	.40
HES	54 (42.8)	21 (48.8)	33 (39.7)	.22
Varicose bleeding	7 (5.5)	2 (4.6)	5 (6)	.55
Acute decompensation	4 (3.1)	0	4 (4.8)	.18
**Etiology of Chronic Liver Disease, n (%)**				
Viral	43 (34.1)	13 (30.2)	30 (36.1)	.32
HBV	34 (26.9)	11 (25.5)	23 (27.7)	.07
HCV	9 (7.1)	2 (4.6)	7 (8.4)	.86
Alcoholic	18 (14.2)	4 (9.3)	14 (16.8)	.19
Cryptogenic	33 (26.1)	15 (34.8)	18 (21.6)	.08
NASH	6 (4.7)	3 (6.9)	3 (3.6)	.33
Autoimmune	3 (2.3)	2 (4.6)	1 (1.2)	.27
PBC	4 (3.1)	1 (2.3)	3 (3.6)	.58
Budd-Chiari	8 (6.3)	2 (4.6)	6 (7.2)	.44
Others	9 (0.7)	1 (0.2)	8 (10)	.83
**Co-existence of HCC, n (%)**	25 (19.8)	0	25 (30.1)	**<.001**
**Comorbidities, n (%)**				
Hypertension	44 (34.9)	15 (34.8)	29 (34.9)	.58
COPD, Asthma	17 (13.4)	6 (13.9)	11 (13.2)	.56
DM	49 (38.8)	22 (51.1)	27 (32.5)	**.04**
Cardiac disorders	31 (24.6)	11 (25.5)	20 (24)	.51
CVD	10 (7.9)	4 (9.3)	6 (7.2)	.46
Chronic renal disease	24 (19)	11 (25.5)	13 (15.6)	.14
Rheumatological	10 (7.9)	3 (6.9)	7 (8.4)	.54
**Source of Infection, n (%)**				
Respiratory System	47 (37.3)	10 (23.2)	37 (44.5)	**.02**
Urinary Tract	32 (25.3)	13 (30.2)	19 (22.8)	.25
SBP	29 (23)	10 (23.2)	19 (22.8)	.57
CRBSI	24 (19)	10 (23.2)	14 (16.8)	.26
Abdominal	12 (9.5)	5 (11.6)	7 (8.4)	.39
Soft tissue	7 (5.5)	2 (4.6)	5 (6)	.55
**Microorganisms, n (%)**				
Gram negative	52 (41.2)	13 (30.2)	39 (46.9)	.06
Gram positive	41 (32.5)	16 (37.2)	25 (30.1)	.27
Fungal	11 (8.7)	2 (4.6)	9 (10.8)	.21
Viral	11 (8.7)	3 (6.9)	8 (9.6)	.44
**RIFLE stage, n (%)**				
Risk	18 (14.2)	8 (18.6)	10 (12.4)	.56
Injury	26 (20.6)	8 (18.6)	18 (21.6)	.45
Failure	39 (30.9)	8 (18.6)	31 (37.3)	**.04**
Loss	4 (3.1)	1 (2.3)	3 (3.6)	.21
**Requirement of MV, n (%)**	78 (61.9)	10 (23.2)	68 (81.9)	**<.001**
**Requirement of RRT, n (%)**	69 (54.7)	16 (37.2)	53 (63.8)	**<.001**
**Blood transfusion, n (%)**	66 (52.4)	19 (44.2)	47 (56.6)	.195
**Albumin replacement, n (%)**	71 (56.3)	23 (53.5)	48 (57.8)	.641

APACHE, Acute Physiology and Chronic Health Evaluation; CLIF-C ACLF, Chronic Liver Failure-Consortium Acute-on-Chronic Liver Failure; COPD, Chronic Obstructive Pulmonary Disease; CRBSI, Catheter-related bloodstream infections; CVD, cerebrovascular disease; DM, diabetes mellitus; HBV, hepatitis B virus; HCC, hepatocellular carcinoma; HCV, hepatitis C virus; HES, hepatic encephalopathy; HRS, hepatorenal syndrome; ICU, intensive care unit; MELD score, model for end-stage liver disease score; MV, mechanical ventilation; NASH, non-alcoholic steatohepatitis; PBS, primary biliary cirrhosis; PSC, primary sclerosing cholangitis; RRT, renal replacement therapy; SBP, spontaneous bacterial peritonitis; SOFA, Sequential Organ Failure Assessment; RIFLE, risk, injury, failure, loss, end-stage.

*Median (25th percentile-75th percentile). **p-value <0.05 was considered statistically significant.

**Table 2.** Baseline Laboratory Findings According to Intensive Care Unit Outcome in Septic Patients with Chronic Liver Disease

	All Patients (n = 126)	Survivors (n = 43)	Non-Survivors (n = 83)	P**
Hb (g/dL)	9 (8-11.2)	8.7 (7.7-10.5)	9.4 (8.1-12)	.16
WBC (/µL)	11 900 (5800-18 200)	7300 (4400-14 000)	14 400 (8500-21 000)	**<.001**
PMNL (/µL)	9800 (4300-16 000)	6100 (2950-11 200)	11 200 (5900-17 100)	**<.001**
Lymphocyte (/µL)	700 (450-1100)	700 (520-1100)	700 (385-1315)	.84
Lactate dehydrogenase (U/L)	394 (257-611)	298 (223-565)	419 (295-628)	**.003**
Platelet (/µL)	88 000 (50000-139 000)	93 000 (50000-136 000)	86 500 (49800-148 000)	.21
INR	1.72 (1.46-2.2)	1.63 (1.21-1.88)	1.76 (1.5-2.44)	**.002**
aPTT	40 (32-54)	39 (29-53)	42 (34-56)	**.04**
C-reactive protein (mg/L)	77 (32-139)	54 (23-97)	89 (46-167)	**.03**
Procalcitonin (ng/mL)	2.2 (0.7-11)	1.9 (0.43-6.9)	2.9 (0.9-15.8)	.47
Blood urea nitrogen (mg/dL)	57 (36-71)	46 (24-71)	58 (42-71)	.15
Creatinine (mg/dL)	1.9 (1.36-3.0)	1.9 (0.8-3.8)	2.2 (1.5-2.9)	.83
Albumin (g/dL)	2.4 (2-2.7)	2.6 (2.1-3)	2.3 (2-2.6)	.29
Aspartate transaminase (U/L)	81 (45-149)	52 (36-110)	89 (60-187)	**<.001**
Alanine transaminase (U/L)	33 (21-71)	26 (18-38)	51 (21-99)	**.003**
Total bilirubin (mg/dL)	3.3 (2.2-8.5)	2.7 (1.8-8.3)	3.6 (2.2-9.1)	.29
Direct bilirubin (mg/dL)	2.0 (1.0-5.2)	1.7 (0.6-3.2)	2.0 (1.1-5.4)	.41
Lactate (mmol/L)	3.8 (2.4-6.7)	2.4 (1.7-3.8)	4.6 (3.3-8.1)	**<.001**
NLR	11 (6.5-23.7)	9 (5-21)	12.5 (7.4-25.5)	**.015**
dNLR	4.7 (3.1-9.4)	4.1 (3-7.5)	4.9 (3.6-11.5)	.07
PLR	93 (59-247)	99 (76-220)	92 (50-287)	.63
PCR	39 (19.4-93.7)	34 (21-120)	39.5 (19-77.1)	.72
LAR	1.6 (1-2.9)	0.93 (0.7-1.6)	2.15 (1.5-3.5)	**<.001**

dNLR, derived neutrophil-lymphocyte ratio; Hb, hemoglobin; LAR, lactate albumin ratio; NLR, neutrophil-lymphocyte ratio; PCR, platelet-creatinine ratio; PLR, platelet-lymphocyte ratio; PMNL, polymorphonuclear leukocytes; WBC, white blood cell.

*Median (25th percentile-75th percentile). **p-value <0.05 was considered statistically significant.

**Table 3.** Multivariate Analysis of Independent Risk Factors for Intensive Care Unit Mortality in Septic Patients with Chronic Liver Disease

	Adjusted OR (95% CI)	P**
APACHE II Score	**1.183 (1.003-1.396)**	**.046**
SOFA Score	1.101 (0.820-1.480)	.521
MELD Score	0.891 (0.762-1.041)	.145
CLIF-C ACLF Score	**1.104 (1.002-1.216)**	**.046**
Child-Pugh Score	1.182 (0.780-1.789)	.430
Respiratory infection as a source of sepsis	0.248 (0.054-1.135)	.072
Co-existence of HCC	0 (0-0)	.998
WBC	1.00 (1.00-1.00)	.168
NLR	0.998 (0.976-1,020)	.872
LAR	**2.992 (1.277-7.009)**	**.012**

APACHE, Acute Physiology and Chronic Health Evaluation; CLIF-C ACLF, Chronic Liver Failure-Consortium Acute-on-Chronic Liver Failure; HCC, hepatocellular carcinoma; LAR, lactate albumin ratio; MELD Score, Model for End-Stage Liver Disease Score; MV, mechanical ventilation; NLR, neutrophil-lymphocyte ratio; RRT, renal replacement therapy; SOFA, Sequential Organ Failure Assessment; WBC, white blood cell. **p-value <0.05 was considered statistically significant.

## Data Availability

The data that support the findings of this study are available on request from the corresponding author.
