# Supplementary material for: Prognostic Value of Inflammatory Markers in Septic Critically Ill Patients with Chronic Liver Disease: A Retrospective Analysis*
Source: Turk J Gastroenterol. 2025 Jun 16;36(9):600–8. doi: 10.5152/tjg.2025.24794 (PMC12432157; doi:10.5152/tjg.2025.24794)
Supplement: Supplementary Material [file supplementary_material.pdf]

Supplementary Table 1. Comparison of baseline characteristics according to ICU outcome in subgroup patients with low CLIF-C ACLF Score

	All Patients (n=55)	Survivors (n=34)	Non-survivors (n=21)	P value**
Age (mean±SD)	55.4±16.3	59±13.2	49±19	**0.042**
Gender, n (%)				**0.054**
Female	22 (40)	17 (50.0)	5 (23.8)	
APACHE II Score (mean±SD)	22.2±6.3	21.0±5.1	25.0±7.2	**0.012**
SOFA Score (mean±SD)	9.2±3.3	8.0±3.1	11.0±3.0	**0.007**
Glasgow Coma Scale*	14 (11-15)	14 (12-15)	10 (7-13)	0.155
Length of ICU stay (day)*	6 (3-11)	5 (3-8)	10 (4-13)	**0.05**
MELD Score*	23 (17-27)	23 (17-27)	23 (17-26)	0.910
ACLF Grade n (%)				**0.011**
I	28 (50.9)	19 (55.9)	9 (42.9)	
II	15 (27.3)	5 (14.7)	10 (47.6)	
III	1 (1.8)	0 (0)	1 (4.8)	
CLIF-C ACLF Score*	47 (39-52)	46 (39-52)	49 (40-53)	0.376
Child-Pugh Score*	10 (8-11)	10 (8-11)	10 (8-12)	
Child-Pugh, n (%)				0.872
A	2 (3.6)	1 (2.9)	1 (4.8)	
B	23 (41.8)	15 (44.1)	8 (38.1)	
C	30 (54.5)	18 (52.9)	12 (52.9)	
Shock on ICU admission, n (%)	20 (36.4)	9 (26.5)	11 (52.4)	**0.05**
Respiratory failure on admission, n (%)	13 (23.6)	5 (14.7)	8 (38.1)	**0.047**
**Complication related to liver disease on admission n (%)**				
HRS	14 (25.5)	9 (26.5)	5 (23.8)	0.826
HES	23 (41.8)	16 (47.1)	7 (33.3)	0.316
Varicose bleeding	4 (7.3)	2 (5.9)	2 (9.5)	0.632
Acute decompensation	1 (1.8)	0 (0)	1 (4.8)	0.382
**Etiology of chronic liver disease, n (%)**				
Viral	18 (32.7)	10 (29.1)	8 (38.1)	0.505
HBV	16 (88.9)	9 (90.0)	7 (87.5)	0.867
HCV	2 (11.1)	1 (10.0)	1 (12.5)	1.00
Alcoholic	6 (10.9)	3 (8.8)	3 (14.3)	0.664
Cryptogenic	17 (30.9)	12 (35.3)	5 (23.8)	0.371
NASH	4 (7.3)	3 (8.8)	1 (4.8)	1.00
Autoimmune	2 (3.6)	2 (5.9)	0 (0)	0.519
PBS	2 (3.6)	1 (2.9)	1 (4.8)	1.00
Budd-Chiari	2 (3.6)	1 (2.9)	1 (4.8)	0.551
Others	4 (7.3)	3 (8.8)	1 (4.8)	0.519
**Co-existence of HCC, n (%)**	5 (9.1)	0 (0)	5 (23.8)	**0.006**
**Comorbidities, n (%)**				
Hypertension	15 (27.3)	12 (35.3)	3 (14.3)	0.089

COPD, Asthma	8 (14.5)	6 (17.6)	2 (9.5)	0.696
DM	22 (40.0)	18 (52.9)	4 (19.0)	**0.013**
Cardiac disorders	10 (18.2)	10 (29.4)	0 (0)	**0.009**
CVD	6 (10.9)	4 (11.8)	2 (9.5)	1.00
Chronic renal disease	11 (20.0)	9 (26.5)	2 (9.5)	0.174
Rheumatological	4 (7.3)	2 (5.9)	2 (9.5)	0.632
**Source of infection, n (%)**				
Respiratory System	17 (30.9)	7 (20.6)	10 (47.6)	**0.035**
Urinary Tract	16 (29.1)	11 (32.4)	5 (23.8)	0.498
SBP	13 (23.6)	8 (23.5)	5 (23.8)	1.00
CRBSI	9 (16.4)	7 (20.6)	2 (9.5)	0.457
Abdominal	7 (12.7)	5 (14.7)	2 (9.5)	0.696
Soft tissue	3 (5.5)	1 (2.9)	2 (9.5)	0.551
**Microorganisms, n (%)**				
Gram negative	20 (37.0)	10 (29.4)	10 (50.0)	0.549
Gram positive	17 (30.9)	12 (35.3)	5 (23.8)	0.13
Fungal	4 (7.3)	2 (5.9)	2 (9.5)	0.632
Viral	5 (9.3)	3 (8.8)	2 (9.5)	1.00
**RIFLE stage, n (%)**				
Risk	14 (25.5)	7 (20.6)	7 (33.3)	0.464
Injury	8 (14.5)	5 (14.7)	3 (14.3)	
Failure	12 (21.8)	7 (20.6)	5 (23.8)	
Loss	1 (1.8)	0 (0)	1 (2.9)	
**Requirement of MV, n (%)**	24 (43.6)	8 (23.5)	16 (76.2)	**<0.001**
**Requirement of RRT, n (%)**	22 (40.0)	12 (35.3)	10 (47.6)	0.365
**Blood Transfusion, n (%)**	29 (52.7)	14 (41.2)	15 (71.4)	**0.029**
**Albumin Replacement, n (%)**	30 (54.5)	16 (47.1)	14 (66.7)	0.156

*median (25th percentile-75th percentile) **p-value <0.05 was considered statistically significant. CLIF-C ACLF: Chronic Liver Failure-Consortium Acute-on-Chronic Liver Failure APACHE: Acute Physiology and Chronic Health Evaluation, SOFA: Sequential Organ Failure Assessment, MELD Score: Model for End-Stage Liver Disease Score, ICU: Intensive Care Unit, HRS: Hepatorenal Syndrome, HES: Hepatic encephalopathy, HBV: Hepatitis B virus , HCV: Hepatitis C virus, NASH: Non-alcoholic steatohepatitis, PBS: Primary biliary cirrhosis, PSC: Primary sclerosing cholangitis, HCC: Hepatocellular carcinoma, COPD: Chronic Obstructive Pulmonary Disease, DM: Diabetes mellitus, CVD: Cerebrovascular disease SBP: Spontaneous bacterial peritonitis, CRBSI: Catheter-related bloodstream infections MV: Mechanical ventilation, RRT: Renal replacement therapy

Supplementary Table 2. Baseline laboratory findings according to ICU outcome in subgroup patients with low CLIF-C ACLF Score

	All Patients (n=55)	Survivors (n=34)	Non-survivors (n=21)	P value**
Hb (g/dL)	8.7 (7.9-10.7)	8.8 (7.9-10.7)	8.7 (8.1-10.2)	0.556
WBC (/µL)	6101 (3910-11790)	5981 (3910-11300)	6650 (4100-13700)	0.678
PMNL (/µL)	4582 (3200-9800)	4320 (2950-9100)	5035 (3245-10650)	0.597
Lymphocyte (/µL)	600 (292-930)	616 (460-1076)	575 (155-768)	0.111
Lactate dehydrogenase (U/L)	379 (235-653)	345 (223-565)	511 (354-1071)	0.060
Platelet (/µL)	74000 (50000-110000)	75300 (51200-108000)	67000 (40700-124300)	0.665
INR	1.57 (1.4-1.88)	1.54 (1.34-1.75)	1.68 (1.34-1.75)	0.060
aPTT	34 (29-43)	34 (29-39)	37 (32-55)	0.110
C-reactive protein (mg/L)	79 (28-126)	67 (27-126)	92 (28-127)	0.563
Procalcitonin (ng/mL)	1.42 (0.46-8.06)	1.16 (0.41-10.03)	2.03 (0.65-7.5)	0.636
Blood Urea Nitrogen (mg/dL)	51 (28-74)	47 (25-71)	54 (34-77)	0.488
Creatinine (mg/dL)	1.7 (0.76-2.5)	1.81 (0.9-3.84)	1.48 (0.74-1.85)	0.111
Albumin (g/dL)	2.5 (2.15-2.8)	2.5 (2.15-2.8)	2.3 (2.18-2.6)	0.349
Aspartate transaminase (U/L)	70 (45-136)	53 (36-110)	110 (70-183)	**0.002**
Alanine transaminase (U/L)	37 (23-78)	26 (21-38)	58 (47-116)	**<0.001**
Total Bilirubin (mg/dL)	2.7 (1.87-4.9)	2.7 (1.8-4.8)	2.9 (2.2-5.24)	0.735
Direct Bilirubin (mg/dL)	1.3 (0.7-2.6)	1.2 (0.7-2.6)	1.5 (1.1-2.53)	0.415
Lactate (mmol/L)	2.9 (2.1-4.4)	2.47 (1.8-3.8)	4.5 (2.9-7.0)	**0.001**
NLR	7.71 (5.2-21.93)	7.1 (5-20)	8.55 (5.95-31)	0.157
dNLR	4.4 (2.95-8.67)	4.14 (2.9-6.55)	4.9 (3.35-12)	0.226
PLR	128 (79.4-259)	98 (76-247)	160 (90-343)	0.311
PCR	40 (18.9-117.2)	32 (20-89)	58.9 (19-131.2)	0.729
LAR	1.3 (0.78-2.05)	0.90 (0.74-1.57)	2.05 (1.3-2.94)	**<0.001**

*median (25th percentile-75th percentile) **p-value <0.05 was considered statistically significant.
dNLR: derived neutrophil-lymphocyte ratio, Hb: hemoglobin, LAR: Lactate albumin ratio, NLR: neutrophil-lymphocyte ratio, PCR: platelet creatinine ratio, PLR: platelet lymphocyte ratio, PMNL: Polymorphonuclear leukocytes, WBC: White Blood Cell

Supplementary Table 3. Multivariate Analysis of Independent Risk Factors for ICU mortality in subgroup patients with low CLIF-C ACLF Score

	Adjusted OR (95% CI)	P Value**
APACHE II Score	1.105 (0.899-1.357)	0.343
SOFA Score	0.931 (0.630-1.376)	0.719
**LAR**	**3.768 (2.120-12.682)**	**0.032**
Alanine transaminase	1.003 (0.990-1.016)	0.657
Gender	3.034 (0.378-24.379)	0.296
**Respiratory infection as a source of sepsis**	**8.689 (1.036-72.839)**	**0.046**
Co-existence of HCC	0 (0-0)	0.999
Blood transfusion	4.659 (0.564-38.511)	0.153

**p-value <0.05 was considered statistically significant.APACHE: Acute Physiology and Chronic Health Evaluation, SOFA: Sequential Organ Failure Assessment, HCC: Hepatocellular carcinoma, LAR: Lactate albumin ratio,

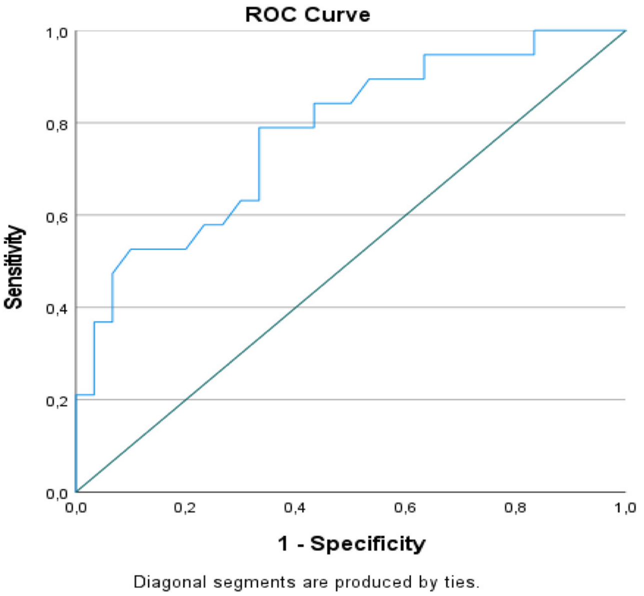

Supplementary Figure 1. ROC Curve of LAR (AUC; Area under the curve, LAR: Lactate albumin ratio, ROC: Receiver Operating Characteristic).
